# Parental Genome Imbalance Causes Hybrid Seed Lethality as Well as Ovary Abscission in Interspecific and Interploidy Crosses in *Nicotiana*

**DOI:** 10.3389/fpls.2022.899206

**Published:** 2022-05-19

**Authors:** Hai He, Kumi Sadahisa, Shuji Yokoi, Takahiro Tezuka

**Affiliations:** ^1^Laboratory of Plant Breeding and Propagation, Graduate School of Life and Environmental Sciences, Osaka Prefecture University, Sakai, Japan; ^2^Laboratory of Breeding and Genetics, Graduate School of Agriculture, Osaka Metropolitan University, Sakai, Japan; ^3^Education and Research Field, School of Agriculture, Osaka Metropolitan University, Sakai, Japan; ^4^Bioeconomy Research Institute, Research Center for the 21st Century, Osaka Metropolitan University, Sakai, Japan

**Keywords:** auxin treatment, endosperm balance number, hybrid seed lethality, interploidy cross, interspecific cross, ovary abscission, tobacco

## Abstract

Enhanced ovary abscission after pollination and hybrid seed lethality result in post-zygotic reproductive isolation in plant interspecific crosses. However, the connection between these barriers remains unclear. Here, we report that an imbalance in parental genomes or endosperm balance number (EBN) causes hybrid seed lethality and ovary abscission in both interspecific and intraspecific-interploidy crosses in the genus *Nicotiana*. Auxin treatment suppressed ovary abscission, but not hybrid seed lethality, in an interspecific cross between *Nicotiana suaveolens* and *N. tabacum*, suggesting that ovary abscission-related genes are located downstream of those involved in hybrid seed lethality. We performed interploidy crosses among *N. suaveolens* tetraploids, octoploids, and neopolyploids and revealed hybrid seed lethality and ovary abscission in interploid crosses. Furthermore, a higher maternal EBN than paternal EBN caused these barriers, as previously observed in *N. suaveolens* × *N. tabacum* crosses. Altogether, these results suggest that maternal excess of EBN causes hybrid seed lethality, which in turn leads to ovary abscission through the same mechanism in both interspecific and interploidy crosses.

## Introduction

Speciation is highly dependent on the evolution of reproductive isolation by the accumulation of barriers to gene exchange (Kulmuni et al., 2020). Reproductive isolation involves various pre-mating and post-mating prezygotic and postzygotic isolating barriers in animals and plants (Coyne and Orr, 2004; Rieseberg and Willis, 2007; Rieseberg and Blackman, 2010). However, post-mating isolation barriers in plants can be obstacles to breeding by interspecific crossings. Conspecific pollen precedence and gametic incompatibility are examples of post-mating prezygotic isolating barriers (Rieseberg and Willis, 2007). In turn, postzygotic isolation barriers include enlarged ovary (immature fruit) abscission (Gupta et al., 1996; He et al., 2019), seed abortion or hybrid seed lethality (Coughlan et al., 2020; Dziasek et al., 2021), hybrid weakness (Shiragaki et al., 2019, 2020), hybrid lethality (Kawaguchi et al., 2021; Si et al., 2021; Tezuka et al., 2021; Mino et al., 2022), and hybrid sterility (Li et al., 2020) in seedlings of the F_1_ generation, as well as hybrid breakdown recognized in generations after the F_1_ (Matsubara et al., 2007; Zhang et al., 2021). These isolating barriers may be observed independently or combined, even in a single cross-combination.

Hybrid seed lethality has been observed in both intraspecific interploidy and interspecific crosses. While the endosperm is generally a triploid tissue and an important component of seeds that supports embryo development and germination, abnormal endosperm development is considered to cause hybrid seed lethality (Oneal et al., 2016; Dziasek et al., 2021; Köhler et al., 2021; Städler et al., 2021), which is often explained by effective ploidy or endosperm balance number (EBN). Further, normal development of the endosperm requires a 2:1 maternal:paternal EBN ratio, a deviation from which results in endosperm developmental failure (Johnston et al., 1980; Coughlan et al., 2020; Städler et al., 2021). In plants with nuclear-type endosperm, inviable hybrid seeds show a disturbance in the timing of endosperm cellularization, which is an important developmental transition for embryo development in this type of endosperm (Ishikawa et al., 2011; Sekine et al., 2013; Lafon-Placette et al., 2017; İltaş et al., 2021). Although few examples of hybrid seed lethality have been reported in plant species with *ab initio* cellular-type endosperm, where karyokinesis and cytokinesis occur simultaneously without syncytial phase (Vijayaraghavan and Prabhakar, 1984; Floyd and Friedman, 2000), inviable hybrid seeds show impaired endosperm proliferation (Oneal et al., 2016; Roth et al., 2018).

Post-pollination ovary or pod abscission has been reported in several interspecific crosses of the genera *Cicer* (Mallikarjuna, 1999), *Lupinus* (Gupta et al., 1996), *Phaseolus* (Mok et al., 1978), and *Vigna* (Barone et al., 1992), in Fabaceae. In these cases, ovarian abscission was accompanied by hybrid seed lethality. Rabakoarihanta et al. (1979) presumed that a severe delay in embryo and endosperm divisions might be the major cause of ovary abscission in *Phaseolus* interspecific crosses, although no direct evidence has been provided.

Recently, we reported ovary abscission occurring in interspecific crosses between the octoploid *Nicotiana suaveolens* accession PI 555565 (♀) and allotetraploid *N. tabacum* (♂), in Solanaceae (He et al., 2019). In this cross, type II seed lethality was also observed with characteristics of precocious developmental transition of the endosperm, subsequent narrowing of the endosperm region as if pressed by surrounding cells, and developmental arrest of embryos in the early globular stage (He et al., 2020). In contrast, the other two accessions of *N. suaveolens* yielded different results after crossing with *N. tabacum*: thus, tetraploid *N. suaveolens* PI 555568 produced normal seeds, whereas octoploid *N. suaveolens* PI 555561 produced abnormal seeds, showing type I seed lethality characterized by precocious developmental transition and subsequent developmental arrest of the endosperm, and abnormal hypertrophy of the embryo during the globular state (the main differences between type I and II seed lethality were the size of the endosperm and the embryo at the globular state). Further, although ovary abscission was not observed in either cross, successive increases in maternal ploidy using ploidy-manipulated lines of PI 555568 and PI 555561 resulted in successive type I and type II seed lethality, and the latter was accompanied by ovary abscission. Therefore, it was suggested that a high maternal-genome excess cross might cause severe seed developmental defects and ovarian abscission (He et al., 2020). However, because this study was based on interspecific crosses, it is unclear whether the cause of abnormal seed development and ovary abscission was only the difference in parental ploidy levels. Furthermore, the cause and result relationship between hybrid seed lethality and ovary abscission also remains unclear.

In this study, we treated peduncles with auxin after pollination to suppress ovary abscission and observed seed development in the interspecific cross between *N. suaveolens* PI 555565 and *N. tabacum* to determine whether ovary abscission is caused by hybrid seed lethality or vice versa, or completely independent isolating barriers occur, because auxin is known to usually inhibit organ abscission (Nakano and Ito, 2013). Additionally, intraspecific-interploidy crosses were conducted using *N. suaveolens* accessions with or without ploidy manipulation to obtain further insight into hybrid seed lethality and ovary abscission in *Nicotiana* interspecific crosses. Our findings demonstrated that maternal genome excess over the paternal genome causes hybrid seed lethality, thereby leading to ovary abscission in both interspecific and interploid crosses.

## Materials and Methods

### Plant Materials

Three *N. suaveolens* accessions, PI 555561 (2n = 8x = 64), PI 555565 (2n = 8x = 64), and PI 555568 (2n = 4x = 32; He et al., 2019), provided by the United States *Nicotiana* Germplasm Collection (Lewis and Nicholson, 2007) and *N. tabacum* ‘Red Russian’ (2n = 4x = 48) provided by the Leaf Tobacco Research Center, Japan Tobacco Inc., Oyama, Japan, were used. We also used chromosome-doubled plants (neopolyploids) of PI 555561 (2n = 16x = 128) and PI 555568 (2n = 8x = 64) developed in a previous study (He et al., 2020), and the PI 555565 (2n = 16x) plants from this study. As previously described (He et al., 2020), chromosome doubling of PI 555565 (8x) was induced using colchicine and was confirmed by flow cytometry using external standardization (Hendrix and Stewart, 2005; Dolezel et al., 2007), where PI 555565 (8x) was used as the standard, against which each sample was analyzed. Next, nuclei were isolated from leaves using Otto buffer (Otto, 1990), stained with 4′,6-diamidino-2-phenylindole (DAPI), and analyzed using a flow cytometer CyFlow Space (Sysmex Partec, Görlitz, Germany; purchased from CytoTechs, Tsuchiura, Japan) and WinMDI 2.9 software.^1^ All plants used for crossing experiments were grown under fluorescent lamps (FL40S·BRN; Toshiba Lighting and Technology Corp., Yokosuka, Japan; approximately 70 μmol m^−2^ s^−1^) in a cultivation room under a 16:8 h light/dark photoperiod regime, at 25°C.

### Crossing Experiments

Conventional crossing was performed as follows: flowers of plants used as maternal parents were emasculated 1 day before anthesis and pollinated with the pollen of the paternal plants. For auxin treatments in crosses between PI 555685 (8x) and *N. tabacum*, lanolin paste containing 0, 10, 100, or 1,000 μM of indole-3-acetic acid (IAA) or 1-naphthaleneacetic acid (NAA) was applied to the peduncles of PI 555565 (8x) at 7 days after pollination (DAP), because the precocious developmental transition of the endosperm was observed at least before 6 DAP and enlarged ovaries of PI 555685 (8x) dropped at 12–17 DAP (He et al., 2019; He et al., 2020); auxin treatments were not conducted before 7 DAP, to avoid possible phytotoxicity expressed as browning of peduncles and ovaries.

One hundred thirty seed weights were determined for interspecific crosses with auxin treatments and intraspecific-interploidy crosses, respectively. An analytical balance (AB54; Mettler Toledo, Greifensee, Switzerland) was used, with three capsules (three replicates) for each cross and seed weights were expressed as single seed weights. For seeds obtained after performing the crosses with auxin treatment, the surface area of seeds was calculated based on photographs of seeds using ImageJ software (Schneider et al., 2012). Seed germinability was evaluated using *in vitro* sowing. Briefly, seeds were soaked in 0.5% gibberellic acid (GA_3_) solution for 30 min and sterilized with 5% sodium hypochlorite for 15 min. Sterilized seeds were sown in Petri dishes (90 mm diameter, 17 mm deep) containing 25 ml of half-strength MS medium (Murashige and Skoog, 1962) supplemented with 1% sucrose, solidified with 0.8% agar (pH 5.8), and then cultured at 28°C for 30 days under continuous illumination (approximately 150 μmol m^−2^ s^−1^).

### Histological Observation

Histological analyses of seeds after pollination were conducted as follows: collected samples were fixed in formalin–acetic acid–alcohol (FAA), after which air in the tissue was extracted using a vacuum pump prior to storing the samples at room temperature until further use. After fixing, samples were dehydrated in an ethanol and t-butyl alcohol series (ethanol:t-butyl alcohol:water = 4:1:5, 5:2:3, 10:7:3, 9:11:0, 1:3:0, and 0:10:0). The t-butyl alcohol was gradually replaced with paraffin at 63°C, over 1 week, in an open bottle to evaporate traces of t-butyl alcohol and then they were embedded in paraffin. Embedded samples were then cut into 10–12-μm-thick sections using a microtome (PR-50; Yamato Kohki Industrial, Asaka, Japan). The tissue slices were placed on glass slides with distilled water (DW) and dried overnight at 50°C on a warming plate. The slides were deparaffinized twice in xylene for 30 min (twice) and hydrated in a graded ethanol series (100%, 95%, 85%, 70%, and 50% in DW). All sections were first treated with 3% iron alum and then stained with 1% fast green (90% ethanol) for 1 min at room temperature. The sections were observed under a microscope (BX50; Olympus) under conventional bright-field illumination. The area of the endosperm was calculated based on photographs of sections using ImageJ software.

### Statistical Analysis

Data were analyzed using the SPSS Statistics software (version 22; IBM, Armonk, NY, United States). Seed weight and surface area were compared using Tukey’s multiple comparison test.

## Results

### Suppression of Ovary Abscission by Auxin Treatment

**Figure 1:**
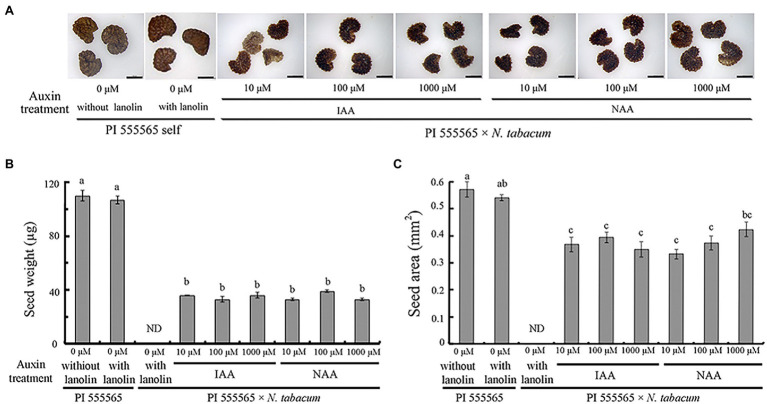
Aborted seeds after auxin treatment in the cross *Nicotiana suaveolens* PI 555565 (8x) × *Nicotiana tabacum*. **(A)** Appearance of normal seeds produced by the self-pollination of PI 555565 and aborted seeds from the interspecific cross. Scale bars = 500 μm. **(B)** Single seed weight. **(C)** Surface area of seeds. Bars represent standard error. Different lowercase letters indicate significant differences at the 5% level as per Tukey’s test. ND, no data.

**Figure 2:**
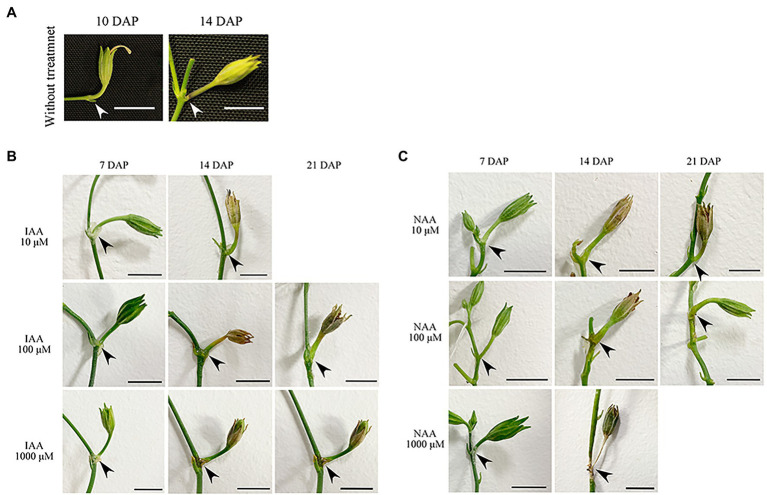
Suppression of ovary abscission by auxin treatment in the cross *Nicotiana suaveolens* PI 555565 (8x) × *Nicotiana tabacum*. **(A)** Ovaries treated without lanolin paste. Enlarged ovaries dropped at 12–17 DAP. **(B)** Ovaries treated with IAA. **(C)** Ovaries treated with NAA. Arrowheads indicate the abscission zone at the intersection between the peduncle and branch. Scale bars = 1 cm. DAP, days after pollination.

**Figure 3:**
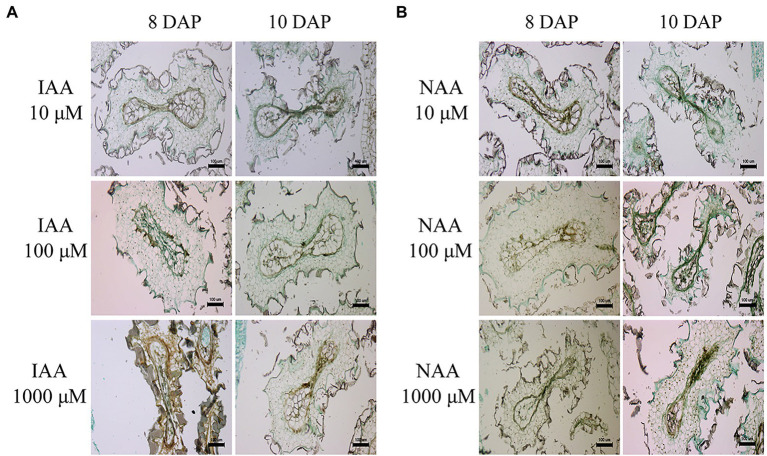
Abnormal endosperm development and characteristics of type II hybrid seed lethality in the cross *Nicotiana suaveolens* PI 555565 (8x) × *Nicotiana tabacum* upon auxin treatment. **(A)** Seeds produced after IAA treatment. **(B)** Seeds produced after NAA treatment. Scale bars = 100 μm. DAP, days after pollination.

To investigate whether ovary abscission was caused by hybrid seed lethality or vice versa in the cross PI 555565 (8x) × *N. tabacum*, peduncles were treated with lanolin paste containing IAA or NAA after pollination. No difference was observed between self-crosses of PI 555565 (8x) treated with or without lanolin paste alone, indicating that lanolin itself did not affect the crossing results (Figure 1; Table 1). In auxin untreated peduncles of the cross PI 555565 (8x) × *N. tabacum*, ovary abscission occurred as expected (Table 1). All IAA and NAA concentrations suppressed ovary abscission and seeds were obtained (Figure 2; Table 1), although they appeared to be empty (Figure 1A); the seeds from the cross PI 555565 (8x) × *N. tabacum* were significantly lighter (33–39 μg) and generally showed a smaller surface area (0.33–0.42 mm^2^) than the seeds of self-pollinated plants of PI 555565 (8x; 107–110 μg and 0.54–0.57 mm^2^ respectively), and never germinated (Figure 1B; Table 1). Furthermore, characteristics of type II seed lethality were observed in the seeds from the cross PI 555565 (8x) × *N. tabacum*; the endosperm region narrowed as if pressed by surrounding cells, and embryos did not develop (Figure 3).

**Table 1:** Effect of auxin treatment on ovary abscission and seed viability in the cross between *Nicotiana suaveolens* PI 555565 (8x) and *Nicotiana tabacum*.

Cross combination	Auxin treatment	No. of flowers pollinated	No. of ovaries		No. of capsules		No. of seeds sown	No. of plants obtained
			Enlarged	Dropped after enlarging	Produced	Showing phytotoxicity		
PI 555565 (8x)	0 μM (without lanolin paste)^a^	21	20	0	20	0	59	48 (81.4%^b^)
	0 μM (with lanolin paste)	20	19	0	19	0	104	85 (81.7%)
PI 555565 (8x) × *Nicotiana tabacum*	0 μM (with lanolin paste)	11	11	11	0	0	–	–
	10 μM IAA	10	10	0	10	0	101	0 (0%)
	100 μM IAA	20	20	0	20	0	82	0 (0%)
	1,000 μM IAA	15	15	0	15	0	120	0 (0%)
	10 μM NAA	13	13	0	13	0	235	0 (0%)
	100 μM NAA	10	10	0	10	0	131	0 (0%)
	1,000 μM NAA	22	22	0	19	3	130	0 (0%)

a The data were previously reported by He et al. (2020).

b Percentage of plants obtained (seed germination percentage).

### Seed Development Observed in Crosses Between 8x and 4x of *Nicotiana suaveolens*

**Figure 4:**
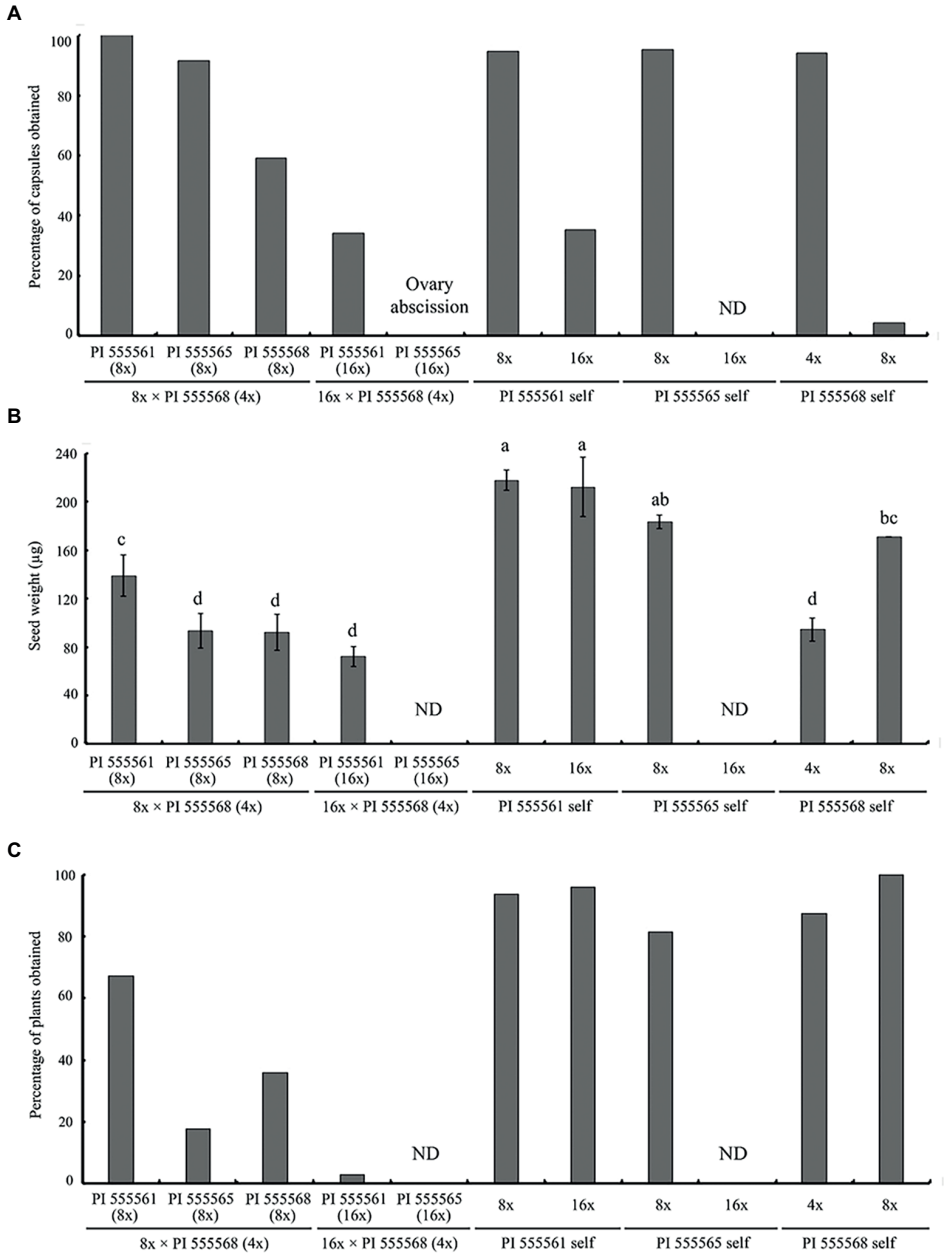
Ovary abscission and seed abortion were observed in intraspecific-interploidy crosses of *Nicotiana suaveolens*. **(A)** Percentages of capsules produced after pollination. **(B)** Single seed weight. Bars represent standard error. Different lowercase letters indicate significant differences at the 5% level as per Tukey’s test. **(C)** Percentages of plants obtained after seed sowing. ND, no data. The data for self-pollinations other than PI 555565 (16x) have been reported previous (He et al., 2020) and are included here for comparison.

Previously, we reported that self-crosses of PI 555568 (4x), PI 555561 (8x), and PI 555565 (8x) yielded capsules at high rates (94.1–95.2%), while PI 555568 (8x) yielded capsules at only 4.2% (He et al., 2020; Table 2). When the three octoploid lines, PI 555561 (8x), PI 555565 (8x), and PI 555568 (8x) were crossed with PI 555568 (4x), abscission of the enlarged ovary was not observed, and 100%, 91.7%, and 59.3% of the flowers produced capsules with seeds in the crosses PI 555561 (8x) × PI 555568 (4x), PI 555565 (8x) × PI 555568 (4x), and PI 555568 (8x) × PI 555568 (4x), respectively (Figure 4A; Table 2).

**Table 2:** Interploidy crosses of *Nicotiana suaveolens*.

Cross combination	No. of flowers pollinated	No. of ovaries		No. of capsules produced	No. of seeds sown	No. of plants obtained	Seed development^c^
		Enlarged	Dropped after enlarging				
PI 555561 (8x) × PI 555568 (4x)	17	17	0	17	131	88 (67.2%^b^)	Normal
PI 555565 (8x) × PI 555568 (4x)	36	33	0	33	142	25 (17.6%)	Normal
PI 555568 (8x) × PI 555568 (4x)	27	16	0	16	100	36 (36.0%)	Normal
PI 555561 (16x) × PI 555568 (4x)	44	15	0	15	112	3 (2.7%)	Type I seed abortion
PI 555565 (16x) × PI 555568 (4x)	11	10	10	0	–	–	Type II seed abortion
PI 555568 (4x)^a^	17	16	0	16	48	42 (87.5%)	Normal
PI 555561 (8x)^a^	19	18	0	18	48	45 (93.8%)	Normal
PI 555565 (8x)^a^	21	20	0	20	59	48 (81.4%)	Normal
PI 555568 (8x)^a^	48	2	0	2	45	45 (100%)	ND
PI 555561 (16x)^a^	34	12	0	12	50	48 (96.0%)	ND
PI 555565 (16x)	17	0	–	–	–	–	–

a The data were previously reported by He et al. (2020).

b Percentage of plants obtained (seed germination percentage).

c Seed development was judged by histological observation.

**Figure 5:**
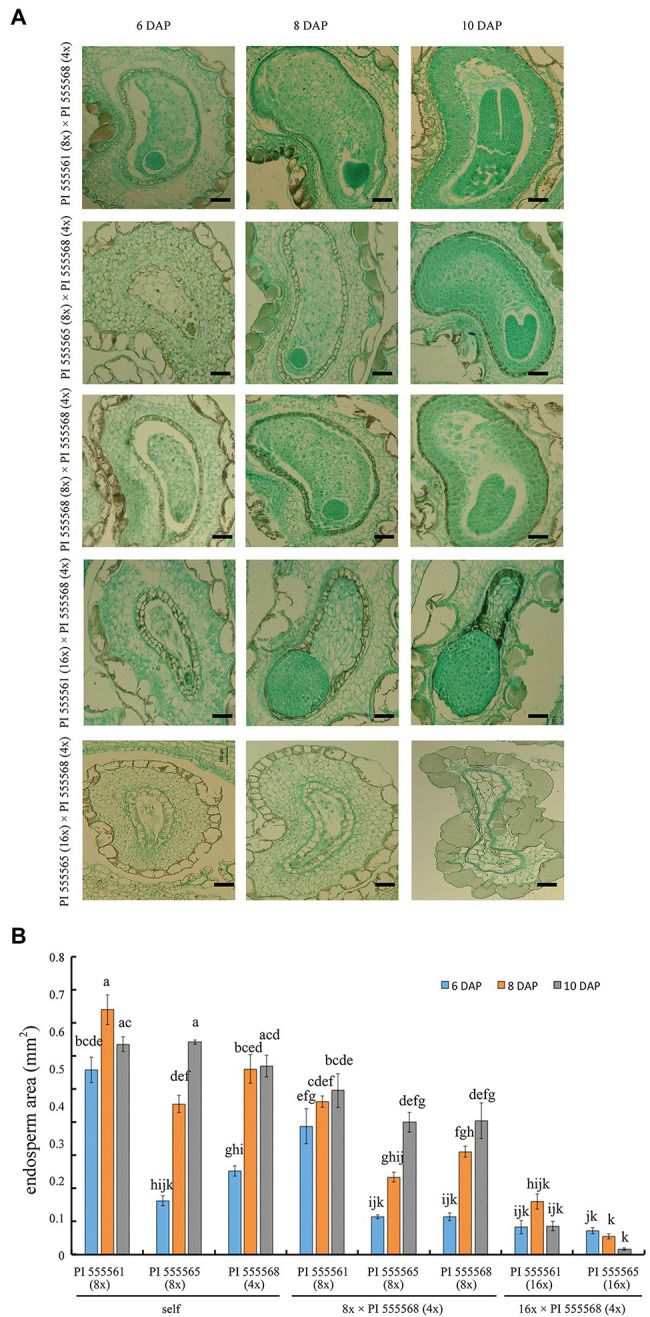
Seed development in intraspecific-interploidy crosses of *Nicotiana suaveolens*. **(A)** Abnormal endosperm and embryo development. Normal endosperm and embryo development were observed in 8x × 4x crosses, while type I and type II hybrid seed lethality were observed in crosses between PI 555561 (16x) or PI 555565 (16x) and PI 555568 (4x), respectively. Scale bars = 200 μm. **(B)** The surface area of the endosperm. Bars represent standard error (*n* = 5 at 6 and 8 DAP, and *n* = 3 at 10 DAP). Different lowercase letters indicate significant differences at the 5% level as per Tukey’s test.

Seeds obtained from the three 8x × 4x crosses were significantly lighter than those of the respective maternal parents (138.9 μg in the interploid cross using PI 555561 vs. 217.8 μg in PI 555561, 93.3 μg in the interploid cross using PI 555565 vs. 183.3 μg in PI 555565, and 92.2 μg in the interploid cross using PI 555568 vs. 171 μg in PI 555568), although seed weights of the 8x × 4x crosses were the same as or higher than those of the paternal parent PI 555568 (4x; 94 μg; Figure 4B). Additionally, the germination rates of the seeds from the three 8x × 4x crosses were lower than those of the respective parental lines; 67.2% in the interploid cross using PI 555561 vs. 93.8% in PI 555561, 17.6% in the interploid cross using PI 555565 vs. 81.4% in PI 555565, 36.0% in the interploid cross using PI 555568 vs. 100% in PI 555568 (8x), and the percentages of all interploid crosses vs. 87.5% in PI 555568 (4x; Figure 4C; Table 2). Further, histological observations showed that an earlier developmental transition of the endosperm occurred at least before 6 DAP in the cross PI 555561 (8x) × PI 555568 (4x), compared with both parents, as well as in the cross PI 555568 (8x) × PI 555568 (4x), compared with the self-cross of PI 555568 (4x) reported by He et al. (2020) (Figure 5A). Moreover, the endosperm region at 6 DAP in the PI 555565 (8x) × PI 555568 (4x) was narrower than that of both parents; 0.23 mm^2^ in the cross PI 555565 (8x) × PI 555568 (4x), whereas 0.45 mm^2^ in PI 555565 (8x) and 0.56 mm^2^ in PI 555568 (4x; Figure 5B). Nevertheless, endosperm and embryo development appeared normal in the three 8x × 4x crosses, i.e., successive stages of embryogenesis, globular, heart-shaped, and torpedo-shaped embryos were observed, as in the cases of self-crosses of the parental lines PI 555568 (4x), PI 555561 (8x), and PI 555565 (8x; Figure 5; Table 2).

### Hybrid Seed Lethality and Ovary Abscission in Crosses Between 16x and 4x *Nicotiana suaveolens* Parents

Neopolyploids (16x) were developed from PI 555565 (8x; Supplementary Figure 1). Using the PI 555565 (16x) and PI 555561 (16x) developed in a previous study (He et al., 2020), crosses between 16x and 4x parents were conducted to investigate the effect of increasing maternal ploidy level on seed development and ovary abscission. In the case of self-pollination, 35.3% of the flowers produced capsules with seeds in PI 555561 (16x; He et al., 2020), whereas none of the flowers produced capsules in PI 555565 (16x; Figure 4A; Table 2). In turn, in the cross PI 555561 (16x) × PI 555568 (4x), 34.1% of flowers produced capsules with seeds. Seed weight was 72.2 μg and significantly lower than that in the self-cross of PI 555561 (16x; 212.2 μg) and crosses PI 555561 (8x) × PI 555568 (4x; Figure 4B). The germination rate of the seeds was 2.7%, which was also lower than that in self-crosses of both parents and the cross PI 555561 (8x) × PI 555568 (4x; Figure 4C; Table 2). As for the cross PI 555565 (16x) × PI 555568 (4x), ovaries were enlarged at a high rate (90.9%) after pollination; however, all enlarged ovaries dropped at 8–14 DAP (Figure 4A; Table 2).

Apparent abnormal seed development was observed histologically in two 16x × 4x crosses (Figure 5). In the cross PI 555561 (16x) × PI 555568 (4x), endosperm development was arrested at 8 DAP, and abnormal hypertrophy of embryos in the globular state as well as void spaces between the seed coat and endosperm were observed from 8 DAP (Figure 5), all of which are characteristics of type I seed lethality. Furthermore, in the cross PI 555565 (16x) × PI 555568 (4x), the endosperm region narrowed as if pressed by surrounding cells, and embryo development was not observed (Figure 5), which are characteristics of type II seed lethality.

## Discussion

### Auxin Suppresses Ovary Abscission but Not Hybrid Seed Lethality

Auxins inhibit the abscission of several types of organs by rendering abscission zone cells insensitive to ethylene, which promotes abscission (van Overbeek, 1952; Nakano and Ito, 2013). Plant growth regulators have been used in Fabaceae to prevent pod abscission in interspecific crosses. Thus, for example, in interspecific crossings of *Phaseolus*, application of NAA alone or in combination with gibberellic acid to the pedicels of pollinated flowers stimulated pod growth and delayed embryo abortion (Al-Yasiri and Coyne, 1964). Similarly, the application of a mixture of gibberellic acid, NAA, and kinetin to the pedicels of developing buds after pollination delayed the abscission of pods and sometimes prevented the abortion of hybrid embryos in interspecific crosses of *Cicer* and *Vigna* (Gosal and Bajaj, 1983; Chen et al., 1990; Mallikarjuna, 1999). In this study, the application of IAA or NAA suppressed ovary abscission in interspecific crosses in *Nicotiana*, confirming the general function of auxin in preventing organ abscission, whether it occurs physiologically or due to an interspecific cross. Previously, we reported that ovarian abscission in interspecific crosses involves a mechanism similar to that of abscission in other organs (He et al., 2020).

Because both ovary abscission and hybrid seed lethality were observed in the cross PI 555565 (8x) × *N. tabacum*, the question arouse as to the cause and the result, or whether completely independent isolating barriers occurred. In a previous study, successive increases in maternal ploidy using ploidy-manipulated lines resulted in successive type I and type II hybrid seed lethality, and the latter was accompanied by ovary abscission. Therefore, we hypothesized that type II seed lethality might lead to ovary abscission (He et al., 2020). Now, this study provided more direct evidence for this hypothesis. Ovary abscission, but not hybrid seed lethality, was effectively suppressed by auxin treatment, strongly indicating that the latter caused ovary abscission in interspecific crosses.

### Hybrid Seed Lethality Leading to Ovary Abscission Is Observed in Interploidy as Well as Interspecific Crosses

Ovary abscission has been reported in interspecific crosses of several genera in Fabaceae (Mok et al., 1978; Rabakoarihanta et al., 1979; Barone et al., 1992; Gupta et al., 1996; Mallikarjuna, 1999) and *Nicotiana* in Solanaceae (He et al., 2020). However, no studies had been conducted on ovary abscission by using intraspecific-interploidy crosses. Additionally, our previous study using *Nicotiana* suggested that a high maternal genome excess might cause type II hybrid seed lethality and ovary abscission (He et al., 2020). Nonetheless, whether these were caused solely due to differences in parental ploidy levels remained unclear. The results of this study on interploid crosses using neopolyploids clearly demonstrated that increases in the ploidy levels of PI 555561 and PI 555565 from 8x to 16x caused type I seed lethality and type II seed lethality, leading to ovary abscission upon crossing with PI 555568 (4x). Thus, the magnitude of the difference in ploidy level between the two parents determines the fate of seeds, namely, whether they will undergo normal development, type I seed lethality, or type II seed lethality, leading to ovary abscission, in interploidy and interspecific crosses of *Nicotiana*.

The histological differences between type I and II seed lethality were in the degree of endosperm and embryo development. For embryos, abnormal hypertrophy and no development at the globular state were observed in type I and II seed lethality, respectively; furthermore, no transition to the heart stage was observed in this case. As embryo development depends on endosperm development (Hehenberger et al., 2012; Lafon-Placette and Köhler, 2014), differences may be caused by deviations from normal progression. Endosperm developmental failure in type I seed lethality occurs before the critical stage for the transition to a heart-shaped embryo. Moreover, whether ovary abscission occurs may also depend on the degree of seed development, as ovary abscission does not occur when the endosperm and/or embryo develops to the level of type I seed lethality, whereas it does occur at the level of type II seed lethality.

### The Severity of Hybrid Seed Lethality in *Nicotiana* Is Explained by the EBN Hypothesis

Endosperm developmental failure responsible for hybrid seed lethality has been well studied in plant genera undergoing nuclear-type endosperm development, such as *Arabidopsis* and *Oryza*. In this developmental mode, a disturbance in the timing of endosperm cellularization is the primary cause observed histologically (Ishikawa et al., 2011; Sekine et al., 2013; Lafon-Placette et al., 2017; İltaş et al., 2021; Köhler et al., 2021). In contrast, endosperm development in *Nicotiana* is an *ab initio* cellular type (Sehgal and Gifford, 1979). The cellular-type endosperm is considered the ancestral type of endosperm development (Floyd and Friedman, 2000). The results of this, and previous studies (He et al., 2020), are consistent with other studies reporting that, although nuclear- and cellular-type endosperms exhibit different developmental abnormalities, endosperm disruption is likely to affect seed development at similar early stages, when embryo development depends on nutrient supply from the endosperm (Roth et al., 2018; Städler et al., 2021).

The EBN hypothesis has been proposed to conceptualize the function of the endosperm in interploidy and interspecific crosses (Johnston et al., 1980). EBN is not directly related to ploidy, and the genome of each plant shows a specific EBN, where a 2:1 maternal: paternal EBN ratio is required for normal endosperm development. According to the EBN hypothesis, the EBN of *N. suaveolens* accessions ranked PI 555565 (8x) > PI 555561 (8x) > PI 555568 (4x). Additionally, the EBN of *N. suaveolens* neopolyploids was as follows: PI 555565 (16x) was the largest, PI 555561 (16x) was the second-largest, and PI 555568 (8x) was similar to or somewhat larger than PI 555561 (8x). Meanwhile, *N. tabacum* would have the same EBN as PI 555568 (4x).

Interestingly, among *N. suaveolens* lineages (this species is endemic to Australia), PI 555565 exhibits a higher EBN than PI 555561, although both accessions are octoploids. Furthermore, although a few anomalies were observed, seed development in 8x × 4x crosses of *N. suaveolens* appeared histologically normal, suggesting flexibility in *Nicotiana* seed development to overcome dosage imbalances. Possibly, evolutionary changes such as sequence changes or duplication of genes might be related to these cases.

Johnston et al. (1980) reported that the EBN of a species may be determined by a few genes rather than the whole genome. To date, many imprinted genes that are expressed in a parent-of-origin-specific manner have been identified as related to hybrid seed lethality (Josefsson et al., 2006; Erilova et al., 2009; Wolff et al., 2015; Florez-Rueda et al., 2016; Garner et al., 2016; Lafon-Placette et al., 2018; Wang et al., 2018; Kinser et al., 2021). Furthermore, transposable elements (Josefsson et al., 2006; Borges et al., 2018) and small RNAs (Erdmann et al., 2017; Martinez et al., 2018; Wang et al., 2018; Satyaki and Gehring, 2019) are reportedly involved in hybrid seed lethality. As for plants undergoing cellular-type endosperm development, a candidate group of genes that may underlie EBN differences was reported in *Solanum* (Roth et al., 2019). However, despite these extensive studies, underlying molecular mechanisms of EBN differences are largely unknown. Therefore, it is an interesting challenge to identify the factors underlying EBN in *Nicotiana* species, which may cause hybrid seed lethality in a dose-dependent manner in interspecific and interploid crosses.

Parental conflict can arise in non-monogamous systems because the interests of maternal and paternal parents can be expected to differ with respect to the amount of maternal resource allocation to the offspring. According to the parental conflict hypothesis, the maternal parent is equally related to all of their progeny and thus should allocate equally, while the paternal parents are only related to their own progeny but not to the competing half-siblings, and thus should somehow direct the maternal parent to allocate differentially (Haig and Westoby, 1989, 1991; Coughlan et al., 2020; Köhler et al., 2021). Thus, co-evolutionary arms race can occur for resource-acquiring paternal alleles and resource-repressive maternal alleles (Coughlan et al., 2020), and reproductive isolation can be established when differences in endosperm or seed development, which are possibly fueled by differences in levels of parental conflict between diverging lineages, reach a critical level. Recent studies have suggested that endosperm-based hybridization barriers are rapidly evolving system of reproductive isolation (Lafon-Placette and Köhler, 2016; Städler et al., 2021). The weak inbreeder/strong outbreeder hypothesis posits that parental conflict is less intense in self-pollinating plants than in outcrossing plants, and thus changes in mating system can change levels of parental conflict (Brandvain and Haig, 2005). Extending this hypothesis, recent studies imply that several factors, such as demographic history, population subdivision, and persistent soil seed banks, as well as mating system changes, can modify effective population size, leading to divergence in EBN (Roth et al., 2019; Coughlan et al., 2020; Städler et al., 2021). Further studies will be needed to verify whether these factors are involved in hybrid seed lethality in *Nicotiana*.

EBN might be related to parental conflict, because opposite phenotypes were observed between seeds obtained from reciprocal crosses of plants with different EBN in *Capsella*, *Mimulus*, and *Solanum*; high EBN × low EBN crosses produce smaller seeds than those in the reciprocal crosses (Lafon-Placette et al., 2018; Roth et al., 2018, 2019; Coughlan et al., 2020). In this and previous studies (He et al., 2020), we conducted maternal excess crosses, but not the reciprocal crosses. For interspecific crosses, this was because fertilization did not occur by conventional cross-pollination, and test-tube pollination in combination with ovule culture was necessary to obtain seeds from crosses between *N. tabacum* and *N. suaveolens* (Tezuka and Marubashi, 2004). However, seeds might be produced by conventional cross-pollination in interploidy paternal excess crosses of *N. suaveolens*, and this should be analyzed in future studies.

## Conclusion

Maternal excess causes hybrid seed lethality based on EBN in *Nicotiana* interspecific and interploid crosses. Ovary abscission occurs depending on the severity of hybrid seed lethality. The endosperm plays an important role in establishing reproductive isolation in angiosperms. *Nicotiana suaveolens* PI 555565 evolved a higher EBN than *N. suaveolens* PI 555561, although both accessions are octoploids. Determining the factors that cause the difference between the two accessions will help to elucidate endosperm-based postzygotic hybridization barriers.

## Data Availability Statement

The original contributions presented in the study are included in the article/Supplementary Material, further inquiries can be directed to the corresponding author.

## Author Contributions

HH and TT conceived and designed the research and wrote the manuscript. HH and KS conducted the experiments. HH, KS, and TT analyzed the data. SY and TT supervised the study. HH prepared the figures. All authors contributed to the article and approved the submitted version.

## Funding

This research was partly supported by JSPS KAKENHI grant numbers JP17K15224 and JP20K05988 from the Japan Society for the Promotion of Science (to TT), and Sasakawa Scientific Research grant numbers 2018-5034 and 2019-5018 from the Japan Science Society (to HH).

## Conflict of Interest

The authors declare that the research was conducted in the absence of any commercial or financial relationships that could be construed as a potential conflict of interest.

## Publisher’s Note

All claims expressed in this article are solely those of the authors and do not necessarily represent those of their affiliated organizations, or those of the publisher, the editors and the reviewers. Any product that may be evaluated in this article, or claim that may be made by its manufacturer, is not guaranteed or endorsed by the publisher.

## Footnotes

^1^ http://www.cyto.purdue.edu/flowcyt/software/Winmdi.htm
