# Supplementary material for: Parental Genome Imbalance Causes Hybrid Seed Lethality as Well as Ovary Abscission in Interspecific and Interploidy Crosses in Nicotiana
Source: Front Plant Sci. 2022 May 19;13:899206. doi: 10.3389/fpls.2022.899206 (PMC9161172; doi:10.3389/fpls.2022.899206)
Supplement: Supplementary file 1 [file Table_1.pdf]

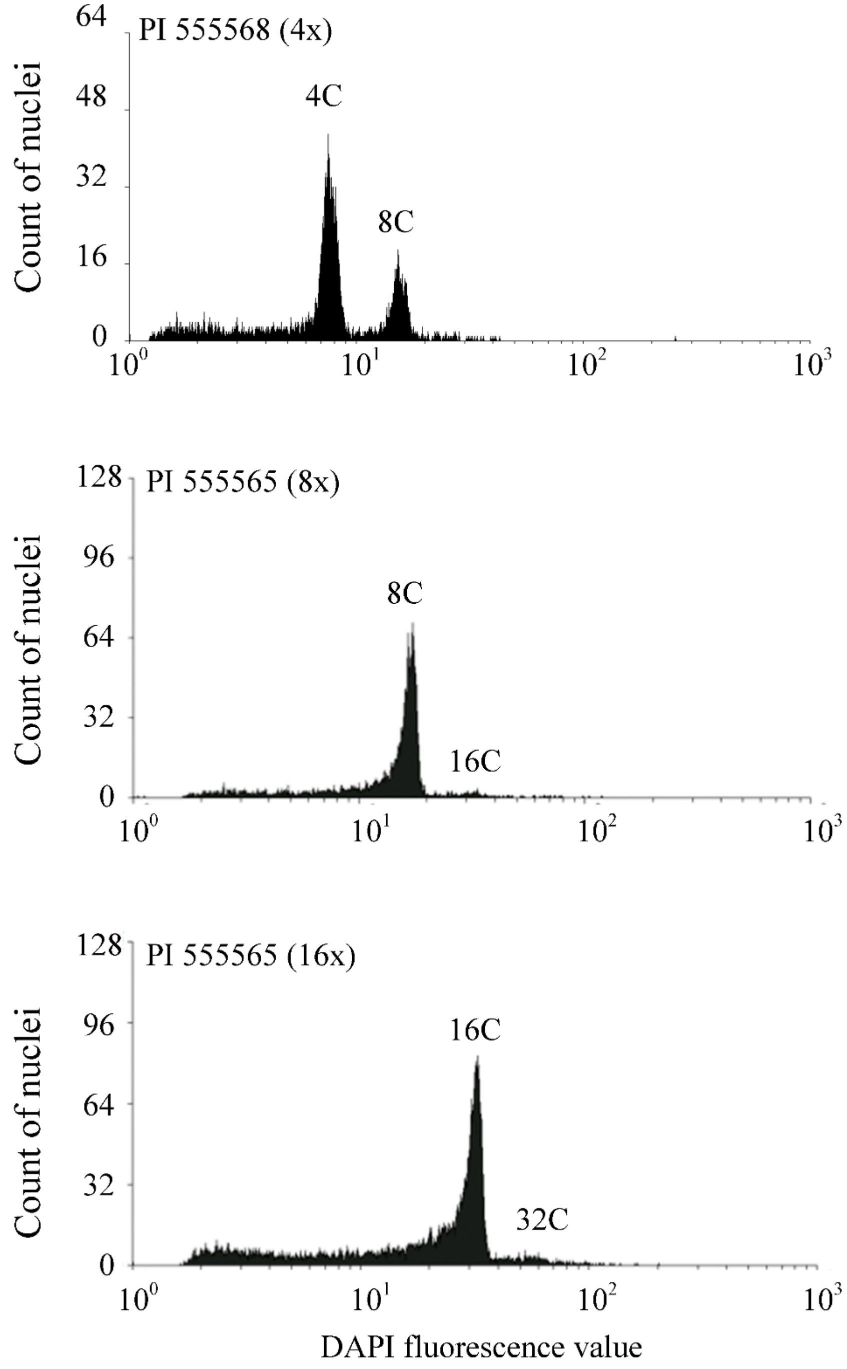

Supplementary Figure 1. Confirmation of the ploidy level in the neopolyploid PI 555565 (16x). Ploidy levels were determined based on the G_1 peak obtained by measuring nuclear DNA content by flow cytometry. PI 555568 (4x) and PI 555565 (8x) were used as external controls.
